# Transpedicular bi‐vertebrae wedge osteotomy in treatment of post‐tubercular spinal deformity: a retrospective study

**DOI:** 10.1186/s12891-021-04220-w

**Published:** 2021-04-12

**Authors:** Yi Huang, Wenhao Hu, Jing Li, Tianhao Wang, Huawei Liu, Guoquan Zheng, Xuesong Zhang, Yan Wang

**Affiliations:** 1 Nankai University School of Medicine, Nankai University, 300071 Tianjin, China; 2 Department of Orthopedics, General Hospital of Chinese People’s Liberation Army, 100853 Beijing, China; 3 Beijing Tsinghua Changgung Hospital, School of Clinical Medicine, Tsinghua University, 102218 Beijing, China

**Keywords:** Post-tubercular Spinal Deformity, Osteotomy, Ultrasonic bone scalpel, Retrospective Study

## Abstract

**Background:**

In the late stage of spinal tuberculosis, the bony destruction and vertebral collapse often leads to significant kyphosis, presenting clinically as a painful gibbus deformity, with increased instability, vertebral body translations and increased risk of neurologic involvement. Spinal osteotomy is thought to be suitable for most patients with severe rigid kyphosis. The aim of this study was to evaluate the efficacy of transpedicular bi-vertebrae osteotomy technique in the patients with Pott’s kyphosis and other post-tubercular spinal deformity.

**Methods:**

Between January 2012 and December 2015, 18 patients with post-tubercular spinal deformity underwent the transpedicular bi-vertebrae wedge osteotomy, with a minimum follow up of 27.0 months. Preoperative and postoperative kyphotic angle, sagittal plane parameters (TK for thoracic deformity, TLK for thoracolumbar and LL for lumbar deformity) and sagittal vertical axis (SVA) were measured. Oswestry Disability Index (ODI), Visual analog scale (VAS) and modified American Spinal Injury Association grading (ASIA) of preoperative and final follow-up were documented and compared.

**Results:**

The average operation time was 305 minutes (range, 200–430 minutes) with a mean intraoperative blood loss of 425 mL (range, 200-700 mL). The kyphotic angles decreased from 80.3° (range, 28.5°-130.8°) preoperatively to 26.1° (range, 7.0°-63.3°) at the final follow-up (*P*<0.01). The mean VAS score was reduced from preoperative 5.2(range, 2-9) to 0.9(range, 0-2, *P*<0.01) and the ODI improved from 55.3% (range, 46%-76%) to 6.3% (range, 2%-18%, *P*<0.01). At final follow-up, there was radiographic evidence of solid fusion at the osteotomy site and fixed segments in all patients. Neurological function improved from ASIA scale D to E in 7 patients, C to D in 3 patients.

**Conclusions:**

Our results suggest that transpedicular bi-vertebrae wedge osteotomy is a safe and effective treatment option for post-tubercular spinal deformity. This technique achieves satisfying correction and fusion rates with adequate decompression of neurological elements.

## Background

Spinal tuberculosis is the most common form of the extra-pulmonary tuberculosis. It accounts for nearly half of the musculoskeletal tuberculosis cases [1]. With or without multidrug chemotherapy, residual kyphosis could present. In the late stage, the bony destruction and vertebral collapse often leads to significant kyphosis, presenting clinically as a painful gibbus, creased instability, and the rate of neurological complications is estimated up to 40 % [2–4].

Anterior approach debridement and fusion known as Hong Kong operation reached a good clinical outcome and was a golden standard for active spinal TB. However, when there is bony destruction of spinal column with kyphosis and instability, posterior instrumentation should be considered to achieve a target sagittal alignment. Anterior, posterior, or combined anterior and posterior procedures that show various degrees of success for correcting kyphosis of TB [5–7].

The late correction of stiff and sharp angular deformities (more than 60°) is only feasible with three-column osteotomies or vertebral column resection (VCR) [3]. When angular kyphosis is located above thoracic spine, there are safety concerns about significant blood loss and morbidity with major complications. Pedicle subtraction osteotomy (PSO), recommended 30–40°as a safe range [8], is usually insufficient to correct severe kyphosis. Wang et al. reported satisfying clinical outcomes and over 65° correction with transpedicular bi-vertebrae wedge osteotomy (TBWO) to manage severe sagittal plane deformity in ankylosing spondylitis [9].

We suppose post-tubercular spinal deformity is a reasonable indication of TBWO [10, 11]. In present study, we evaluate the clinical and radiographic patient results to estimate the efficacy of this technique applying on post-tubercular deformity.

## Methods

### Study design

Patients with post-tubercular spinal deformity admitted to our department from January 2012 to December 2015 were included. Diagnosis was made based on radiographic examination, laboratory tests and histopathology. This study was conducted with approval from the Ethics Committee of Chinese PLA General Hospital and was performed in accordance with the Declaration of Helsinki. Written informed consent was obtained from all participants. And for participants below 18 years, informed consents were obtained from legal guardian. The indications for surgery were as follows: (1) low back pain refractory to conservative treatment; (2) being not able to lie down in dorsal position; (3) increasing neurological deficit. Patients with active infection and who cannot tolerate surgery due to poor cardiopulmonary function were excluded. Anteroposterior, lateral spine radiographs, CT 3-D reconstruction, Magnetic Resonance Imaging (MRI) were available for all patients.

Standing anteroposterior and lateral radiographs were performed preoperatively, postoperatively, and at the time of every follow-up. Lateral radiography was processed with Surgimap (Nemaris Inc., New York, NY, USA). Local and global sagittal parameters were measured and analyzed including kyphotic angle, thoracic kyphosis (TK), thoracolumbar kyphosis (TLK) and sagittal vertical angle (SVA). Kyphotic angle was measured between the maximally tilted upper and lower end vertebrae over the post-tubercular kyphosis by using the standard Cobb’s method. Thoracic kyphosis was defined as the angle between the superior endplate of T1 and the inferior endplate of T12 (for there are upper thoracic deformity above T5). Thoracic lumbar kyphosis was defined as the angle between the superior endplate of T10 and the inferior endplate of L2. Lumbar lordosis was measured between the superior endplate of L1 and S1, and kyphosis is defined as negative. The assessments were performed by two independent assessors.

All patients’ morbid segments, osteotomy levels, instrumented levels, operative time and blood loss were noted. Neurologic deficits were assessed according to the American Spinal Injury Association (ASIA) grading system. Pain was assessed using the visual analogue score (VAS). Disability status was assessed using the Oswestry Disability Index (ODI). Radiological and clinical records were recorded preoperatively, postoperatively and during the last follow-up period.

### Operative technique

All surgeries were performed under monitoring of somatosensory-evoked potentials, transcranial motor-evoked potentials, and free-running electromyography. Under general anesthesia, the patient was placed prone on the operating table, and a standard posterior middle incision was made at the predetermined level. The spine was exposed by dissection lateral to the costotransverse joint at the thoracic level and transverse process of the lumbar. The segmental vessels were coagulated using electric cauterization and hemostatic gauze. Pedicle screws (Weigao Orthopedic,Shandong, China)were then placed three levels above and below the damaged vertebral body by freehand technique. C-arm fluoroscopy was used to confirm the appropriate insertions.

**Fig. 1:**
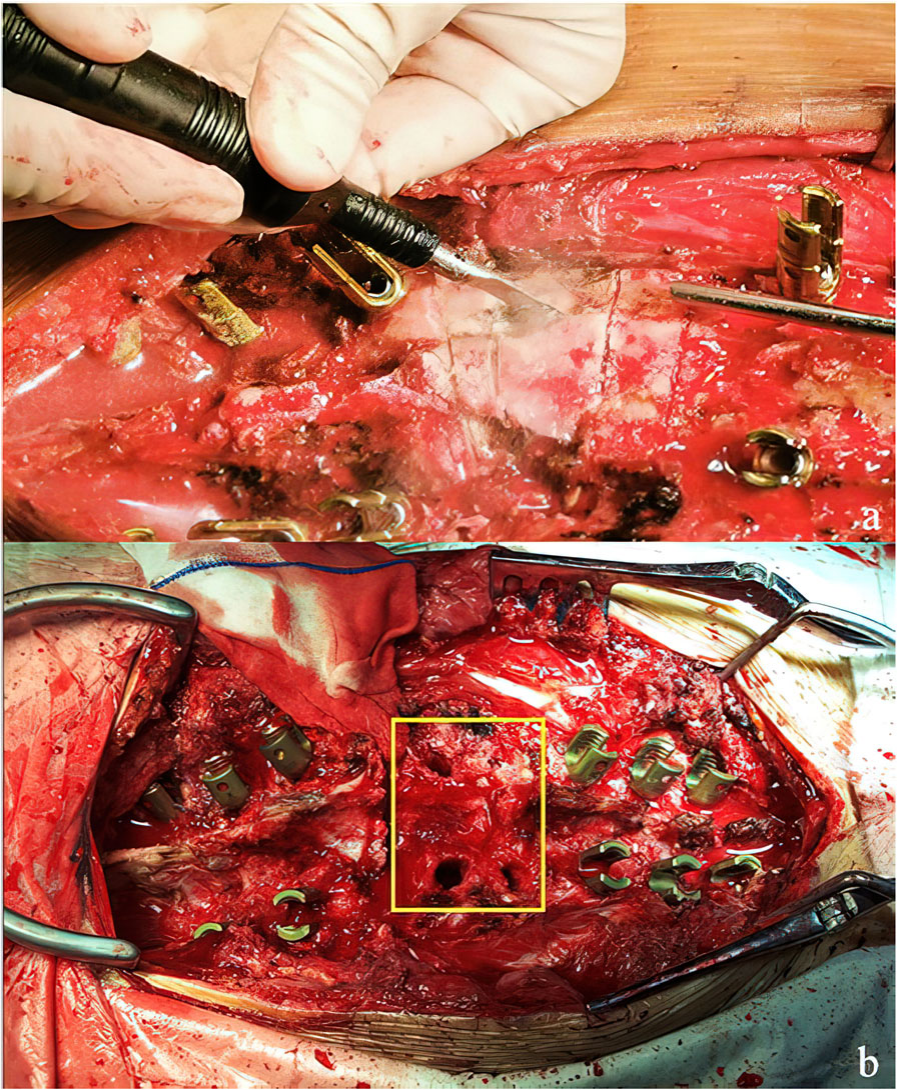
**a** Posterior elements were removed with ultrasonic bone scalpel. **b** The pedicle holes (yellow frame) of the malformed vertebras were created and enlarged utilizing the probe and curette

With ultrasonic bone scalpel (UBS, Weigao Orthopedic,Shandong, China), posterior elements of targeted levels including the spinous process, bilateral lamina, transverse process, and the adjacent facet joints were removed. (Fig. 1a) The spinal cord and nerve roots were then meticulously exposed and protected. The pedicle probe and curette were used to create and enlarge the pedicle holes of the fused malformed vertebras with both sides of the pedicles. (Fig. 1b) Gelatin sponges were placed into the holes for hemostasis. Then posterior parts of pedicles were removed and the spinal canal was opened from both lateral sides. For thoracic segments, nerve root ligation shall be performed if there is a high tension in dura sac. Transverse processes and rib heads could be resected at their bases if there is difficulty in lateral removal. For malformed vertebrae which containing more than 2 pairs of pedicles, through relatively larger pedicle holes, wedge osteotomies were performed in the same manner as designed preoperatively.

**Fig. 2:**
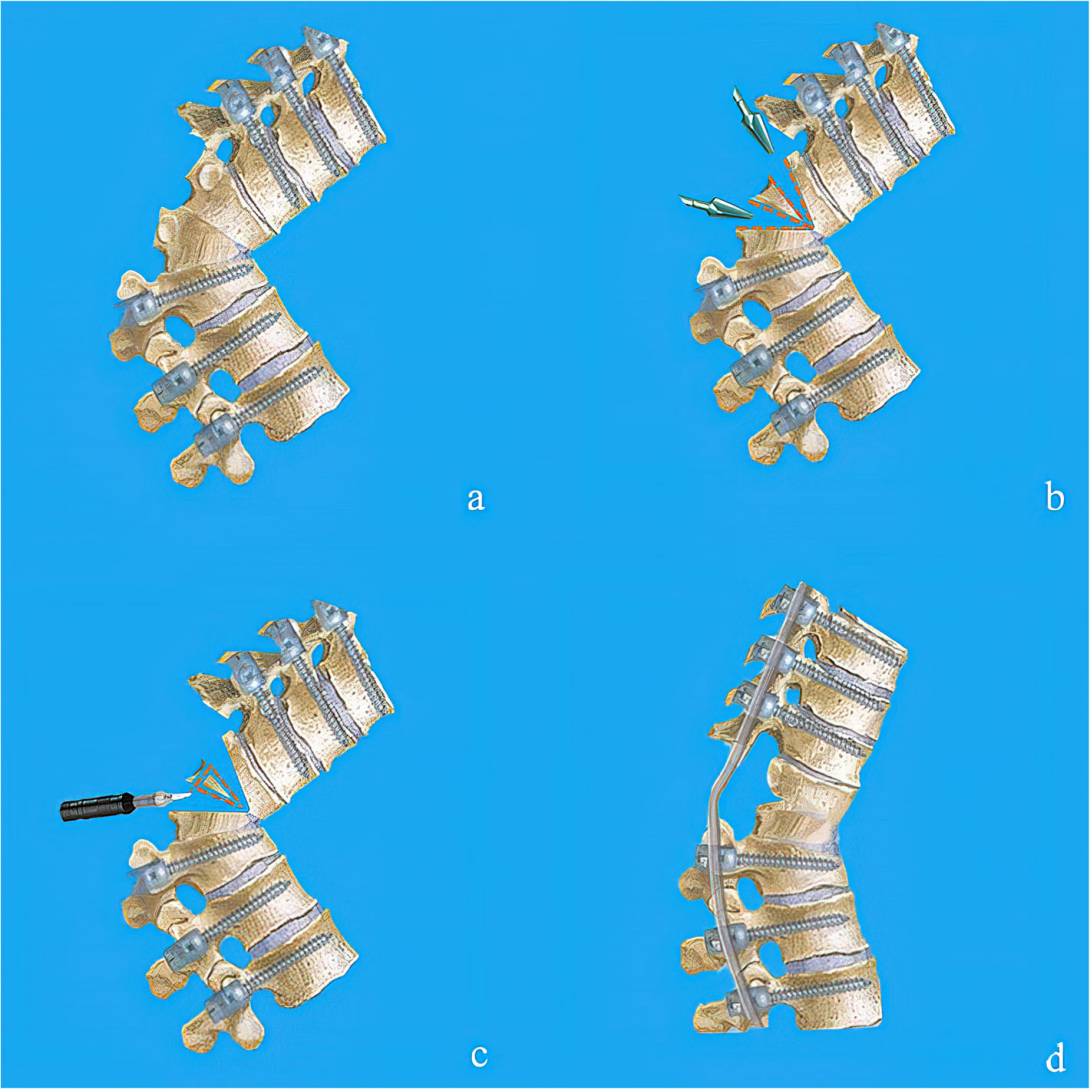
**a** Pedicle screws were placed and posterior elements were removed with ultrasonic bone scalpel. **b** Posterior part of pedicles were removed and the spinal canal was opened laterally. **c** Wedge osteotomies were performed as designed preoperatively including lower and upper endplate and relic of disc between them. **d** After removing the posterior cortical bone of the osteotomised vertebra, the kyphotic spine is corrected using gentle manual force stabilized by a temporary rod

The cancellous bone of the inferior-posterior aspect of upper vertebra and the superior-posterior aspect of lower vertebra one were resected to create a cavity with ultrasonic bone scalpel. Then the ultrasonic bone scalpel was used to make thinning of the irregular anterior cortex and lateral walls. After removing the posterior cortical bone of the osteotomized vertebra, the kyphotic spine is corrected using gentle manual force stabilized by temporary rods. The operating table and the position of the patient were adjusted for the correction. (Fig. 2) Finally, Cantilever procedure was performed to close the osteotomy site, and pressure on the pedicle screws above and below the osteotomy with pre-contoured rods was performed to strengthen the stability. Each step of correction was monitored by somatosensory-evoked potentials, transcranial motor-evoked potentials, and free-running electromyography.

After confirmation of absent soft or bony compression, posterolateral bone grafting was conducted and drainage tube was placed in the surgical field with the wound closed in layer sequence.

### Statistical analysis

All statistical analysis was performed with SPSS v19.0 software (SPSS Inc., Chicago, Illinois). Students’ t-test was used for all analyses, and a *p*-value < 0.05 was considered statistically significant.

## Results

In all, 18 consecutive patients of whom (11 males, 7 females; mean age 37.3 years) with post-tubercular spinal deformity were included. Demography, morbid segments, osteotomy levels, instrumented levels, operative time, blood loss and follow-up time are shown in Table 1. The post-tubercular kyphotic deformities were located at thoracic region in 7 patients, thoracolumbar region in 8 patients and lumbar region in 3 patients. Among all the 18 patients, 15 patients underwent an initial debridement without fusion during childhood. TBWO was performed in all patients. The average operative time was 305 min (range, 200–430 min) with a mean intraoperative blood loss of 425 mL (range, 200–700 mL). All patients completed follow-up of 31.4 months on average, from 27 to 36 months.

**Table 1:** Demographic and Clinical Data

Patient	Age	Sex	Morbid Segments	Osteotomy Levels	Instrumented Levels	Operative Time(min)	Blood Loss(ml)	Follow-up(mon)
1	11	M	T7-9	T8,T9	T5-7,T9-11	290	700	32
2	31	M	T2-4	T2,T3	C5-6,T1,T5-8	430	700	32
3	42	M	T7-9	T8,T9	T5-7,T9-12	340	400	35
4	31	M	T2-6	T3,T4	T1-6	200	400	29
5	21	F	T2-3	T3,T4	T1-2,T4-6	200	200	34
6	41	M	T3-7	T4,T5	T1-3,T7(R),T8-11	260	500	27
7	32	M	T5-8	T5,T6	T1-4,T8-11	400	400	32
8	56	M	L2	L2,L3	T11-L1,L3-4	260	300	29
9	64	F	T11-12	T11,T12	T8-10,L1-3	340	200	33
10	21	M	T8-11	T9,T10	T5-8,T11-L2	430	600	31
11	44	F	T9-12	T10,T11	T7-10,T12-L3	240	500	30
12	41	M	T9-11	T10,T11	L1(R),L2,T7-9,T11	200	300	32
13	15	M	T9-L3	T10,T11	T9-L3	240	300	31
14	48	F	T11-L3	T10,T11	T8-10,L1-4	400	600	34
15	47	F	T10-12	T11,T12	T7-10,T12-L3	230	250	32
16	38	F	L3-4	L3,L4	L1-3,L5-S1,S2(L)	230	400	28
17	65	M	T12-L3	T12,L1	T4-11,L4-S1	420	300	36
18	24	F	L1-4	L3,L4	T8-L1,L4-5	380	600	29

The preoperative and final follow-up data of the 18 patients were shown in Table 2. The kyphotic angles decreased from 80.3°(range, 28.5°–130.8°)preoperatively to 26.1°(range, 7.0°–63.3°)at the final follow-up (*P* < 0.01). TK (T1-T12) of thoracic type kyphosis decreased from 59.3° (range, 25.4°–95.8°) to 31.3(range, 0.3°-47.3°), with a mean correction rate of 55.6 %. TLK of thoracolumbar type kyphosis decreased from 61.5° (range, 29.0°–72.5°) to 17.7° (range, 0.4°–40.0°) with a mean correction rate of 69.0 %. LL angle of lumbar type kyphosis decreased from − 55.6° (range, -70.5°–-36.0°) to 5.4° (range, 2.0°–10.1°) with a mean correction rate of 84.3 %.

**Table 2:** The preoperative, postoperative and last follow-up data

	Type	Sagittal plane parameters (TK/TLK/LL)		SVA		Kyphotic angle(°)		Correction(°)	Correction Rate	Mean Correction Rate	ASIA		ODI(%)		VAS	
Patient	T*,TL*,L*	Pre-	Post-	Pre-	Post-	Pre-	Post-				Pre-	F-follow	Pre-	F-follow	Pre-	F-follow
1	T	25.4	30.8	-4.2	3.2	78.5	34.0	44.5	56.7%	55.6%	C	D	76	12	7	1
2		95.8	42.7	0.1	1.6	77.0	11.3	65.7	85.3%		C	D	68	8	2	0
3		62.3	35.8	1.75	4.7	110.2	63.3	46.9	42.6%		D	E	48	2	3	1
4		57.9	47.3	-7.9	0.5	50.5	30.2	20.3	40.2%		C	E	62	18	2	0
5		30.6	19.3	5.5	-1.7	28.5	15.3	13.2	46.3%		B	C	62	6	2	0
6		61.5	0.3	4.5	2.5	127.6	40.1	87.5	68.6%		C	D	52	6	4	1
7		81.3	42.9	-1.2	0.1	98.0	49.2	48.8	49.8%		D	E	46	2	9	2
8	TL	67.0	25.0	0.5	3.0	67.0	21.0	46.0	68.7%	69.0%	D	E	48	6	8	1
9		29.0	24.0	-4	3.1	31.7	10.0	21.7	68.5%		E	E	46	4	9	2
10		61.6	2.0	-0.8	-0.2	84.0	22.3	61.7	73.5%		D	E	60	6	6	2
11		72.5	0.4	5.2	3.1	85.0	14.6	70.4	82.8%		D	E	52	10	7	2
12		66.0	8.0	1.0	2.9	80.6	23.1	57.5	71.3%		D	D	52	6	7	1
13		68.6	27.3	-6.4	-1.8	54.4	30.9	23.5	43.2%		E	E	50	4	2	0
14		59.5	40.0	8.5	3.3	130.8	48.0	82.8	63.3%		D	E	52	6	3	0
15		67.8	14.5	-5.9	3.9	70.5	13.4	57.1	81.0%		E	E	48	2	7	1
16	L	-36.0	10.1	11.5	-0.8	68.9	10.6	58.3	84.6%	84.3%	D	D	76	10	6	1
17		-60.2	2.0	12.5	4.5	106.0	26.0	80.0	75.5%		D	E	52	4	3	1
18		-70.5	4.0	32.7	1.0	95.6	7.0	88.6	92.7%		E	E	46	2	7	1

The neurological status of the patients was ASIA E in 4 patients (22.2 %), ASIA D in 9 patients (50.0 %), ASIA C in 4 patients (22.2 %) and ASIA B in one patient (5.6 %) preoperatively. A total neurological complication rate is 11.1 %. Neurological events occurred in 2 of the 18 patients with neural monitoring changing intraoperatively, while one regained the signals after standard procedures. One patient suffered transient partial neurological deficit (quadriceps paralysis) post-operatively and resolved completely after 3 months. Dural tears with transient cerebrospinal fluid leakage were encountered in 1 case (5.6 %) who underwent an initial debridement, the tear was covered intraoperatively by muscle and fat grafts, lumbar drainage was placed and removed after seven days. No instrumentation related complication and no deep wound infection were identified.

**Fig. 3:**
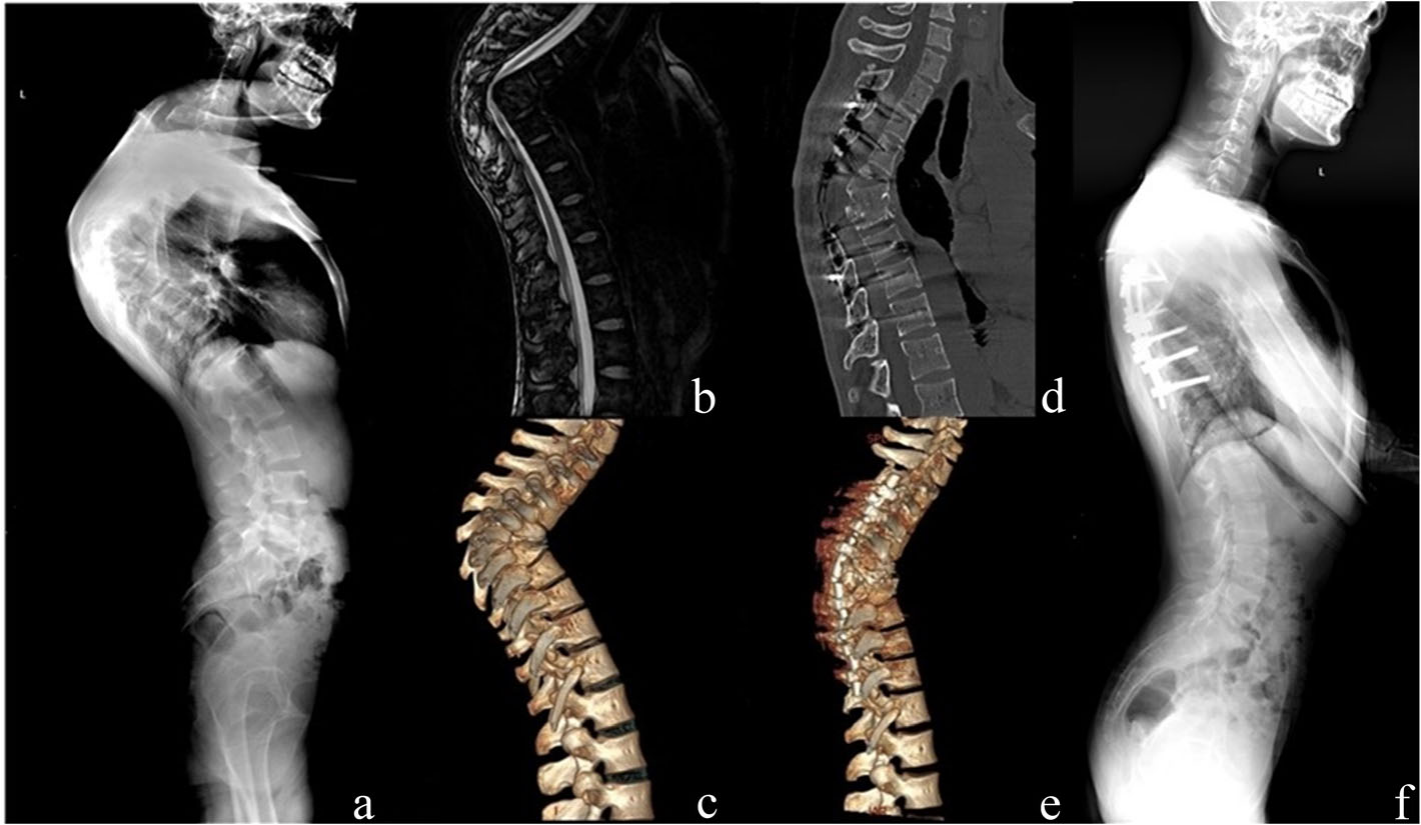
**a**, **b**, **c**. A 32 years old male patient presented with lower limb numbness and mobility restricted. T5-8 spinal column collapsed and deformation, kyphotic angle was 98.0°and SVA was measured − 1.2 cm preoperatively. **d**, **e**, **f**. The transpedicular bi-vertebrae wedge osteotomy was performed in T5 and T6. A correction of 48.8° was achieved in upper thoracic region safely. The patient improved to ASIA E after 6 months, and solid fusion was observed in final follow-up

**Fig. 4:**
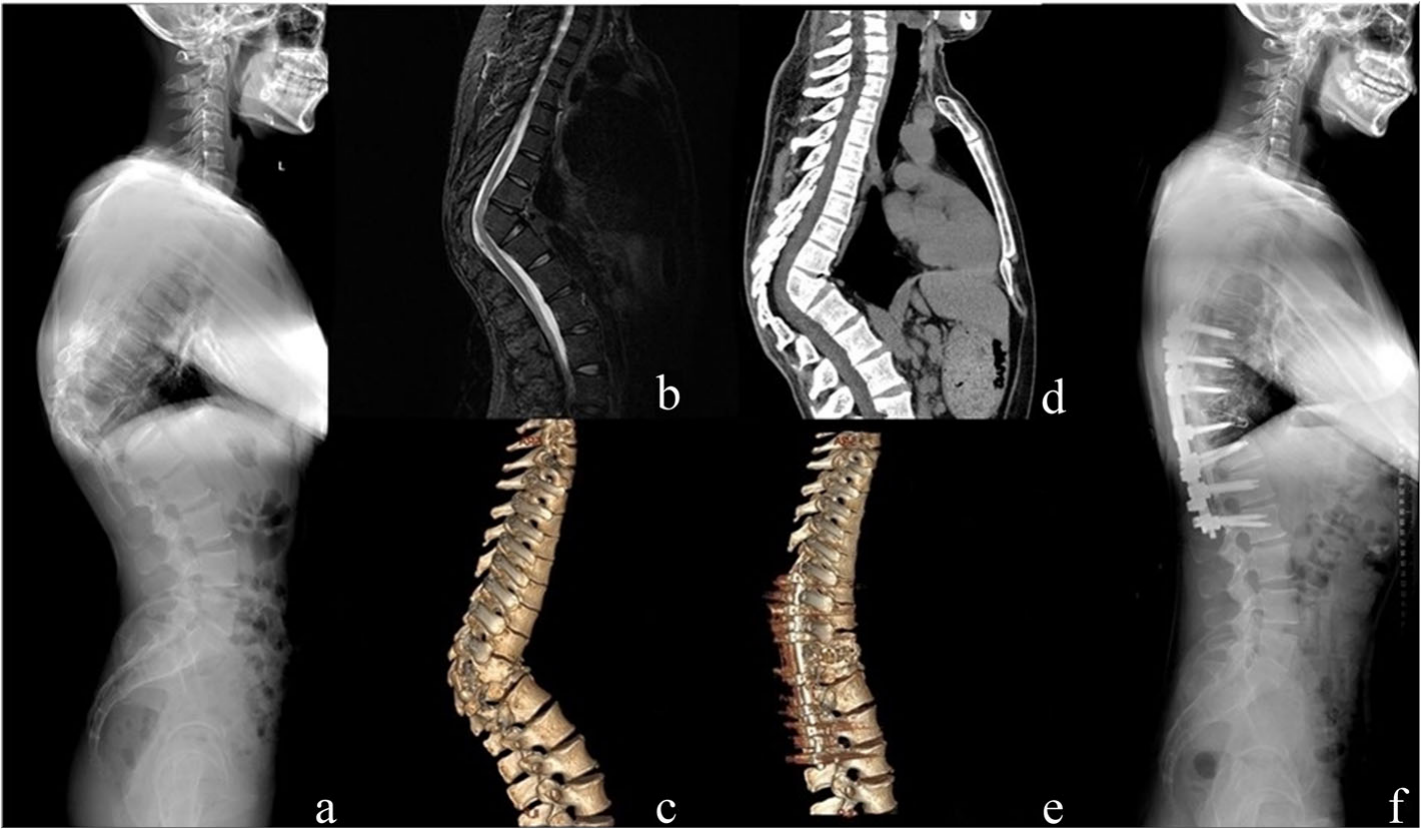
**a**, **b**, **c**, **d**. A 41 years old male patient, presenting with severe back and radicular pain, without cord compression symptom. Anterior column collapse in lower thoracic region. The kyphotic angle of T9-11 was 80.6° and SVA was measured 1.0 cm preoperatively. **e**, **f**. The transpedicular bi-vertebrae wedge osteotomy was performed in T9 and T10. A postoperative kyphotic angle of 23.1°and better sagittal alignment were achieved. Notably, an extra cage was placed in lower intervertebral space to keep height of it

The mean VAS score was reduced from preoperative 5.2 (range, 2–9) to 0.9 (range, 0–2, *P* < 0.01) and the ODI improved from 55.3 % (range, 46–76 %) to 6.3 % (range, 2–18 %, *P* < 0.01). At final follow-up, there was radiographic evidence of solid fusion at the osteotomy site and fixed segments in all patients. (Figures 3 and 4) Neurological function improved from ASIA scale B to C in 1 patient, C to D in 3 patients and D to E in 7 patients at the final follow-up.

## Discussion

TB spondylitis is still a challenging problem in both developing and developed countries [12], which can lead to a significant bone destruction and collapse of the vertebral bodies, resulting in hyper-kyphosis and tethering of the spinal cord [3, 11].For patients with neurological deficits, spinal instability or severe kyphotic deformity, surgery should be considered [3]. The sharp angular hyper-kyphosis often requires complex three-column osteotomies. Currently, the one-stage posterior approach is most often used for minimizing the risk of injury to anterior vascular and visceral structures [13]. Pedicle subtraction osteotomy (PSO), the most popular osteotomy technique, has been applied for progressive tubercular thoracic and thoracolumbar kyphosis. Kalra et al. [10] used pedicle subtraction osteotomy to treat patients with healed tuberculosis of the spine and a resultant kyphosis. The osteotomy is described as closing wedge osteotomy and correction of the deformity is achieved by the shortening of posterior column. However, the technique should be limited to 30°–40° as a safe range of single segment osteotomy; otherwise, the spinal cord is excessively shortened and distorted [14]. Some modifications of PSO are reported that could obtain a greater correction angle without postoperative complications [15]. Wu SS et al. [16] claimed that they obtain a maximum correction angle of 60° at a single level. However, this modified procedure is not suitable to correct a severe kyphotic deformity with a kyphotic angle beyond 90°.

Although Posterior VCR provides adequate amount of surgical correction when compared to all other spinal osteotomy types [17], it is restricted owing to its inevitable neurological risk related to the instability induced during correction of the malformation [18, 19]. The complication rate has been estimated as high as 59 % for posterior VCR [20]. Zheng et al. [21] described Posterior-only bilevel modified vertebral column resection for extremely severe Pott’s kyphotic deformity, and the spinal sagittal kyphotic angle was corrected from a preoperative kyphosis 100.3° to a postoperative angle of 15.9° in their study. This procedure, however, was recommend to be performed at or below lower thoracic spine for security concerns. Transpedicular bi-vertebrae wedge osteotomy (TBWO) was first applied for fixed sagittal deformity of ankylosing spondylitis by Wang et al. [9], and they achieved an average correction of 65.2°. Same technique was applied in Zhao’s research and achieved a 76.8 % correction of the sagittal imbalance [22]. It should be noticed that TBWO could effectively reconstruct regional alignment than many other posterior osteotomy techniques, with a postoperative TK of 31.3°, TLK of 17.7° and LL of 5.4°. And this technique allows spacious room for neural elements and direct vision during osteotomy procedure, thus it can be safely applied in cervical or cervicothoracic area.

For those patients with post-tubercular spinal deformity, we prefer the TBWO in several aspects. First, most post-tubercular fused vertebras were angular deformity, which were treated as one targeted vertebra so that one-level osteotomy could obtain satisfactory outcomes, so it could reasonably reduce operation time and blood loss. The maximal correction angle was 88.6° in lumbar spine (44.3° per segment), which is consisting with previous study. The average operative time was 305 min with a mean intraoperative blood loss of 425 ml. Without corpectomy of deformed vertebrae, correction could be gained from transpedicular procedure and osteotomy site closure, which is time saving and manageable for spine surgeon. Secondly, collapse of anterior cortex of the osteotomy vertebra facilitates the correction of a rigid kyphosis [23], and larger bony contact surface could provide better fusion rates and additional spinal stability. In our study, the average correction rate was 84.3 % in lumbar spine, 69.0 % in thoracolumbar spine and 55.6 % in thoracic spine. The relative low rate of correction in thoracic spine could owning to restriction of the rib cage, but we would not recommend over correcting angular kyphosis in the thoracic spine with high risk of complications such as hemopneumothorax and spinal cord shock. When performing decompression and electrocoagulation in thoracic spine, it is noteworthy that artery of Adamkiewicz is located at this region. Preoperative angiogram is mandatory if the target vertebra located at T7 to L2. And we avoid rhizotomy if a preoperative angiogram has not been performed. During TBWO procedure, consideration should be given during electrocautery near the thoracic foramen or nerve dissection at the left side. However, literature has shown that the artery can be ligated safely as long as the collateral circulation is preserved [24], and we hardly have experienced a vascular spinal cord injury in our center. But as the literature mentioned, it remains a theoretical risk. Thirdly, TBWO could allow greater correction angles for its sufficient decompression of posterior elements, especially for upper thoracic and cervical segments. Neurologic complication is one of the main risks of extensive kyphotic correction by posterior osteotomy. TBWO procedure started with a decancellization procedure along the pedicles. Then osteotomized segments were gently closed with cantilever procedure under monitoring of somatosensory-evoked potentials, transcranial motor-evoked potentials, and free-running electromyography. When angular kyphosis deflexed, dura sac expanded and folded backwards, without obvious axial shortening. the distance of osteotomy closure could larger than substantial dural sac shortening distance. Based on the experience of our center, < 12 mm of substantial posterior shortening distance could be a safe range of shortening and central canal enlargement is critical. The perioperative neurological complication rate is 11.1 % in our study, with 2 of the 18 patients having neural monitoring changing intraoperatively. One patient regained the signals after standard procedures. One quadriceps paralysis was observed and recovered 3 months later postoperatively. No permanent neurological deficit due to TBWO procedure were observed. And there was one dura tears case with no postoperative infection. All of the 18 patients with post-tubercular spinal deformity who underwent TBWO achieved steady fusions with satisfactory rehabilitation. It should be noticed that several of 18 patients had initial poor neurological status and relative younger age, which indicated TBWO procedure demands rather strict patient selection.

In a decade ago, high-speed drill was commonly used in bone removing. However, when close to dura and nerve roots, a downward pressure of drill could be dangerous and disastrous neurological complications could happen. Especially in angular kyphosis, dura was stretched and high-tension, which closely clung to bony structure, and epidural space is extremely narrow. UBS applied in spine surgery has a huge advantage in time saving, minimizing soft tissues injury and could precisely finish bone cutting [25–27]. Utilization of UBS in osteotomy procedure can be safe and compatible in transpedicular bone removing, and leaves a smooth surface, which give less chance of dura tear and nerve injury. However, further research is needed to investigate the impact of UBS heat effect during osteotomy on bony fusion.

To best of our knowledge, there is no literature further discuss the subtype of post-tubercular kyphotic deformity. Our study firstly reviewed three different types of post-tubercular kyphotic deformity. And we carried out the point that a same technique managing post-tubercular kyphotic deformity in different spinal region with different correction ability. Our study had not compared the loss of correction and pelvic morphology. And as the number of the cases are small and this is a single center study, the larger sample research is needed for further study.

## Conclusions

Our results suggest that TBWO technique is a safe and effective treatment option for post-tubercular spinal deformity. This technique achieves satisfying correction and fusion rates with adequate decompression of neurological elements.

## Data Availability

The patients’ data were collected in the Chinese PLA General Hospital. The datasets generated and/or analyzed during the current study are not publicly available due to limitations of ethical approval involving the patient data and anonymity but are available from the corresponding author on reasonable request.

## Abbreviations

TB: Tuberculosis
TBWO: Transpedicular bi-vertebrae wedge osteotomy
VCR: Vertebral column resection
PSO: Pedicle subtraction osteotomy
TK: Thoracic kyphosis
TLK: Thoracolumbar kyphosis
SVA: Sagittal vertical angle
UBS: Ultrasonic bone scalpel
ASIA: American Spinal Injury Association grading system
VAS: Visual analogue score
ODI: Oswestry Disability Index
CT: Computerized tomography
MRI: Magnetic Resonance Imaging
